# Metastatic lymph nodes of occult breast cancer show very low internal echoes

**DOI:** 10.1016/j.radcr.2024.06.068

**Published:** 2024-07-18

**Authors:** Nozomi Uozumi, Shoji Oura

**Affiliations:** Department of Surgery, Kishiwada Tokushukai Hospital, 4-27-1, Kamori-cho, Kishiwada-city, Osaka 596-8522, Japan

**Keywords:** Breast cancer, High Ki-67 labelling index, Occult breast cancer, Very low internal echoes

## Abstract

A 62-year-old woman showed an elevation of carcinoembryonic antigen (CEA) level 15 years after the left breast cancer, i.e., tubule forming type luminal micro invasive cancer, operation. Positron emission tomography/computed tomography (PET/CT) showed avid radio-tracer uptake in her right axillary and supraclavicular lymph nodes. Mammography, ultrasound (US), PET/CT, and magnetic resonance imaging showed no abnormalities in her right breast. US of the enlarged lymph nodes showed very low internal echoes. Pathological study using a core needle biopsy specimen of a right axillary node showed human epidermal growth factor receptor type 2 (HER2) positive atypical cells growing in solid and trabecular fashions. Under the presumed diagnosis of right occult breast cancer, the patient received anti-HER2 agents-containing chemotherapy, leading to marked shrinkage of the enlarged lymph nodes with normalization of the elevated CEA level. To confirm the pathological efficacy, sentinel plus sampling node biopsy was done to the patient. Postoperative pathological study of the resected nodes showed fibrosis and no viable cancer cells. The patient further received radiotherapy both to the right breast and suprarclavicular region followed by adjuvant anti-HER2 agents therapy, and has been well without any recurrences for 39 months. Diagnostic physicians should note that metastatic lymph nodes of occult breast cancer show very low internal echoes.

## Introduction

Occult breast cancer is defined as a pathological condition detected by its axillary lymph node metastasis without any breast cancer visible on imaging in the breast [[1], [2], [3]]. However, magnetic resonance imaging (MRI) has made it possible to depict the very small tumor, unable to be detected on conventional image modalities, in the vast majority of occult breast cancers [[4], [5], [6]].

Ultrasound (US) of metastatic lymph nodes shows similar internal echoes to those of primary breast cancers. Therefore, breast cancer with high internal echoes develops hyper-echoic lymph node metastasis. Conversely, breast cancer with low internal echoes will have hypo-echoic lymph node metastasis.

We herein report a human epidermal growth factor receptor type 2 (HER2)-positive presumed occult breast cancer which was detected by metastatic lymph nodes with very low internal echoes and was successfully treated with neoadjuvant chemotherapy [7] followed by adjuvant radiotherapy to the breast.

## Case report

**Fig. 1:**
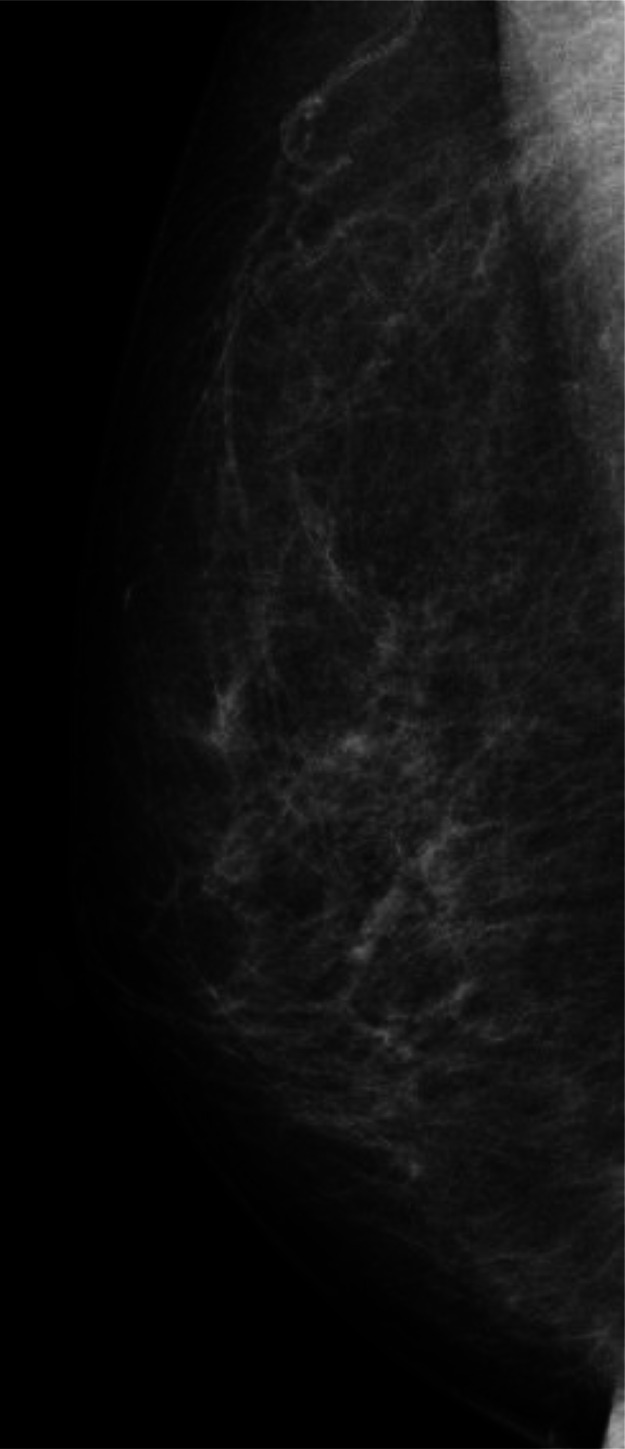
Mammography findings. Medio-lateral-oblique view mammogram with an almost entirely fatty density showed no masses in the right breast.

**Fig. 2:**
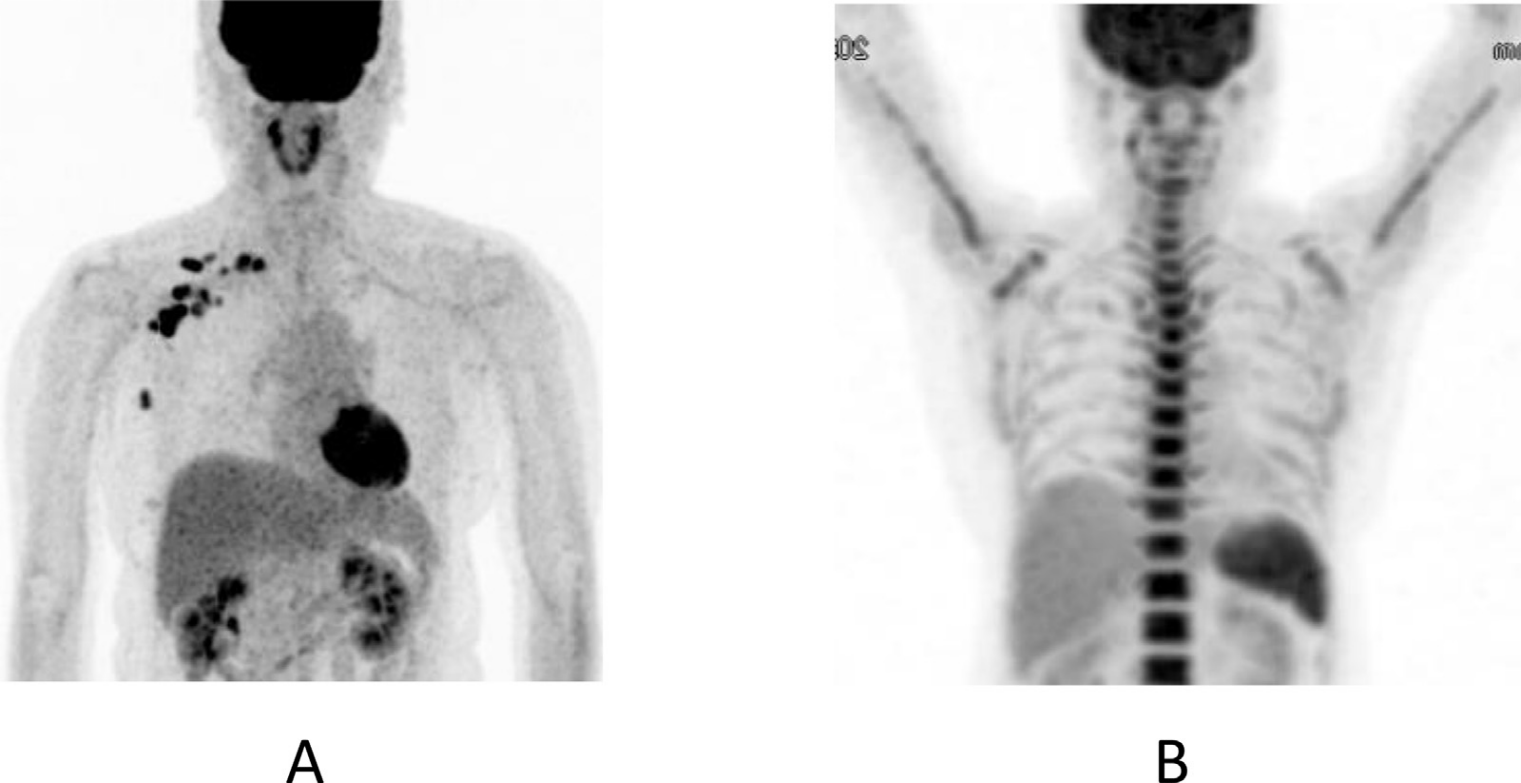
Positron emission tomography/computed tomography (PET/CT) findings. (A) PET / CT showed multiple avid radio tracer uptake in the right axillary and supraclavicular lymph nodes before systemic therapy. (B) PET / CT after systemic therapy showed intense radio tracer uptake in the bones due presumably to the administration of pegfilgrastim on chemotherapy and no uptake in the regional nodes.

**Fig. 3:**
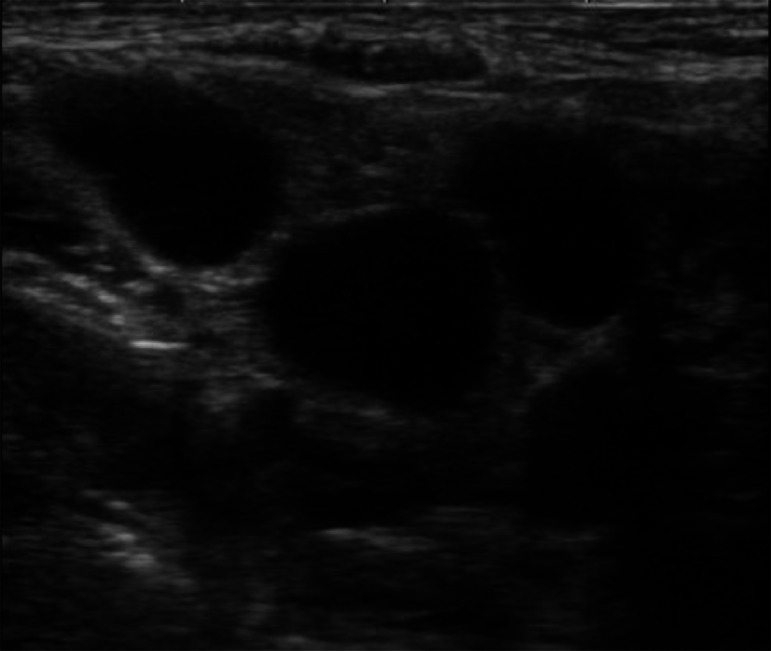
Ultrasound (US) findings. US showed enlarged oval lymph nodes with very low internal echoes in the right supraclavicular region.

**Fig. 4:**
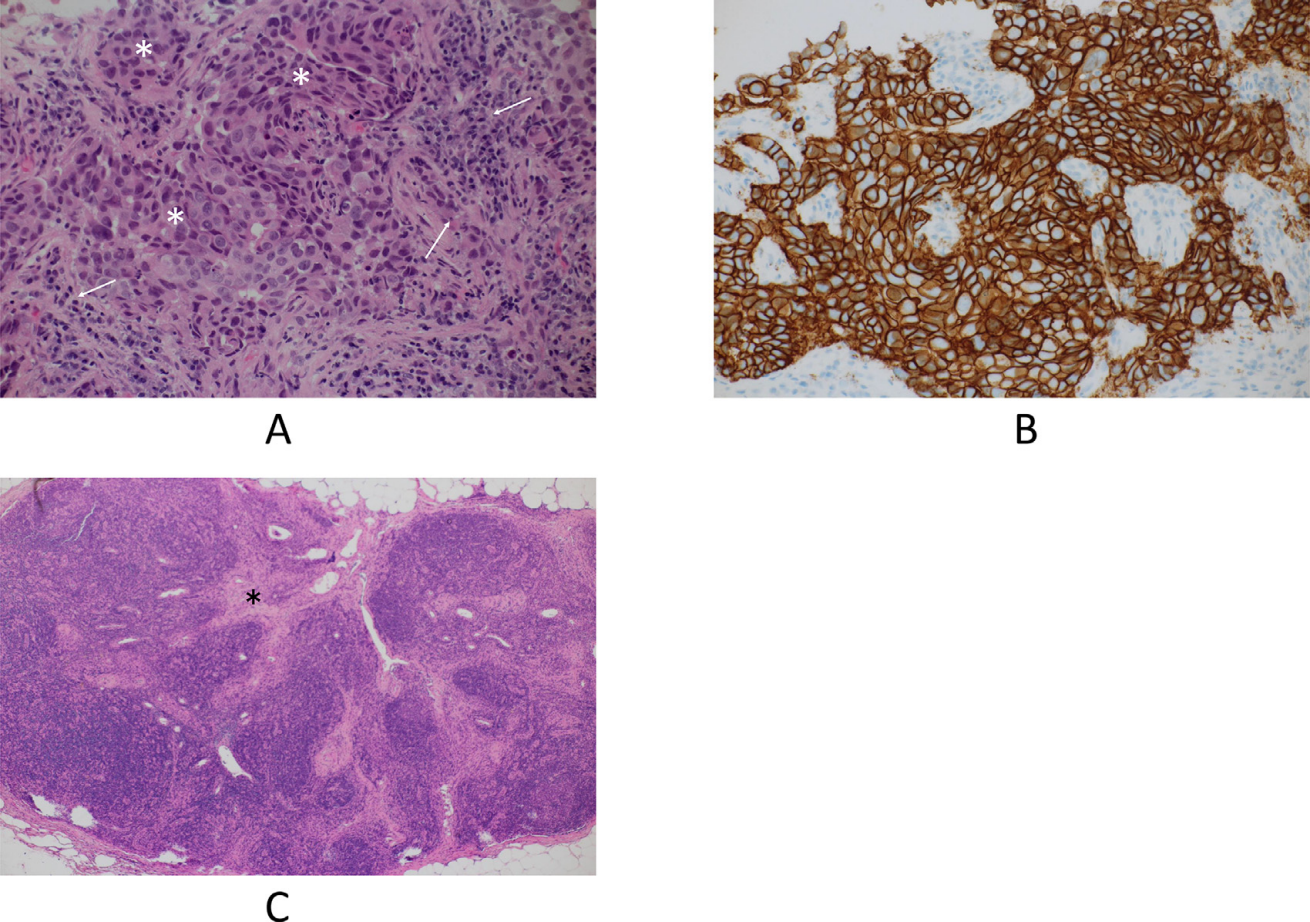
Pathologic findings. (A) Core needle biopsy of a right axillary lymph node pathologically showed atypical cells growing in solid (asterisks) and trabecular fashions (arrows). (B) Immunostaining showed the membranes of the atypical cells to be strongly positive for human epidermal growth factor receptor type 2. (C) Resected lymph nodes had fibrosis (asterisk) and no viable cancer cells.

A 62-year-old woman had undergone breast-conserving therapy and sentinel node biopsy 15 years before. Her left breast cancer was micro invasive cancer, i.e., invasive components less than 1 mm, and was a tubule forming phenotype. After the standard 10 years of follow-up period with 5 years' adjuvant tamoxifen therapy, the patient had continued annual check-ups for breast cancer mainly with mammography and tumor marker evaluations. Despite the normal mammography findings (Fig. 1), an elevated carcinoembryonic antigen (CEA) level of 31.3 ng/mL made the patient receive a recurrence check with positron emission tomography/computed tomography (PET/CT). PET/CT showed avid radio tracer uptake both in the right axillary and supraclaicular lymph nodes, i.e., a maximum standard uptake value of 8.46 (Fig. 2A), but not in the mammary gland. US showed enlarged lymph nodes with very low internal echoes (Fig. 3) and no breast masses. The attending breast surgeon, therefore, recommended endocrine therapy to the patient under the judgement of late recurrence of her left breast cancer. The patient, however, was referred to our hospital because she was not convinced of the diagnosis of late recurrence. Pathological study using a core needle biopsy specimen of a right axillary node showed atypical cells growing in solid and trabecular fashions (Figs. 4A and B) with estrogen receptor negativity, progesterone receptor negativity, human epidermal growth factor receptor type 2 (HER2) positivity, and a Ki-67 labelling index of 35%. Even MRI, however, unfortunately depicted no tumors in her right breast. Therefore, under the diagnosis of presumed HER2-positive occult breast cancer, the patient received 4 courses of trastuzumab, pertuzumab, and docetaxel chemotherapy as a primary systemic therapy and got marked shrinkage of the enlarged lymph nodes (Fig. 2B) with normalization of the elevated CEA level. The patient thereafter underwent sentinel plus sampling node biopsy to evaluate the pathological efficacy of the primary systemic therapy. Pathological study showed fibrosis and no viable cancer cells in the resected lymph nodes (Fig. 4C). The patient received adjuvant radiotherapy both to the right breast and supraclavicular region followed by adjuvant trastuzumab and pertuzumab therapy for additional 9 months. The patient has been well without any recurrences for 39 months.

## Discussion

It is not so rare that breast cancer recurs after the standard follow-up period of 10 years [8]. Her attending physician, therefore, had recommended endocrine therapy to the patient under the diagnosis of late recurrence of her left breast cancer due to the lack of cancer images in her right breast. However, regardless of the time of recurrence, metastasis only to the contralateral axillary and supraclavicular lymph nodes itself is extremely rare [9].

It is well known that the amount of US wave back scattering determines the internal echo levels of the mass. The more the back scattering of US waves is, the higher the internal echoes of the tumor become. Back scattering of US waves increases mainly when the tumor is composed of 2 or more cellular components with different acoustic impedances [10] or when some kind of papillary structures are present in the tumor. We could not get US images of her left breast cancer, naturally being unable to comparatively evaluate the US images between her left breast cancer and right axillary and supraclavicular lymph nodes. However, we could get a pathological report that her left breast cancer had been a micro-invasive breast cancer with a tubule forming phenotype, i.e., one of papillary structures. It, therefore, seemed extremely rare for her breast cancer to cause any distant metastases. In addition, even if her left breast cancer had spread to the contra lateral axillary and supraclavicular lymph nodes, the internal echoes of the enlarged lymph nodes should have been high.

Very low internal echoes imply little back scattering of US waves due to the uniformity of acoustic impedance among mass-constituting cells, suggesting high proliferating nature of the tumor cells. Multiple lymph node metastases caused by image undetectable small tumor also indicates extremely aggressive nature of the tumor. Pathological study of the metastatic lymph node actually showed HER2 positivity and a high Ki-67 labelling index of 35%. Internal echoes, therefore, should be very low without exception in the metastatic lymph nodes of occult breast cancer.

MRI, unfortunately, could not show any abnormality in her right mammary gland in this case. Naturally, it is impossible to determine what therapeutic effect preoperative chemotherapy had on the primary tumor. However, judging from the clinical and pathological efficacy of the anti-HER2 agents-containing chemotherapy against metastatic regional nodes, we can speculate that the systemic therapy had highly brought about complete pathological response also to the primary cancer. Adjuvant radiotherapy to the right breast, therefore, should be judged as an appropriate therapeutic alternative to mastectomy in this case due to no recurrences in the right breast for more than 3 years.

## Conclusion

Breast oncologists should diagnose and treat breast cancer with a thorough understanding of both image formation mechanisms and disease-specific characteristics.

## Patient consent

Written informed consent was obtained from the patient for the publication of this case report and any accompanying images.

## Author contribution

NU designed the concept of this study. SO drafted the manuscript.
